# Spatial relationship between land development pattern and intra-urban thermal variations in Taipei

**DOI:** 10.1016/j.scs.2020.102415

**Published:** 2020-11

**Authors:** Wan-Yu Shih, Sohail Ahmad, Yu-Cheng Chen, Tzu-Ping Lin, Leslie Mabon

**Affiliations:** a Department of Urban Planning and Disaster Management, Ming-Chuan University, Taiwan; b GCRF Centre for Sustainable, Healthy and Learning Cities and Neighbourhoods (SHLC), Urban Studies, School of Social and Political Sciences, University of Glasgow, United Kingdom; c Taiwan Building Technology Center, National Taiwan University of Science and Technology, Taiwan; d Department of Architecture, National Cheng Kung University, Taiwan; e Scottish Association for Marine Science, Scotland, United Kingdom

**Keywords:** Urban heat island effect, Local climate zones, Land surface temperature, Urban planning, Urban geometry, Climate change adaptation

## Abstract

This paper assesses the influence of land development patterns on intra-urban thermal variation in a densely-developed subtropical city, considering joint effect from greenspace pattern and built-up geometry. Despite growing research on urban climates, research at a scale that can support urban planning with scientificallyinformed strategies is still not as well documented for warm climate cities as for temperate cities. In response, this paper uses land surface temperature and geoinformation to assess the subtropical city of Taipei, Taiwan. Results show cooler environments are not only associated with natural surfaces, but also their interrelation with different spatial arrangement of buildings. An open layout tends to have lower temperature at low- to mid-rise buildings, whereas a compact layout is the coolest form for high-rise buildings. Cooling benefit from open layouts is, however, related to an increase in greenery. Clustering distribution of greenspaces produces more notable cooling. Accordingly, this paper proposes four heat mitigation strategies for Taipei: 1) increasing the amount of water bodies and vegetation, with greater coverage and coherence; 2) taking building height and shadow into account during regeneration/development; 3) increasing spacing and greenery between low- to midrise buildings; and 4) avoiding construction of compact low-rise buildings with corrugated iron steel.

## Highlights

• Surface temperature varies with building height, spacing, materials and greenness.
• Low-rise factories made of corrugated iron steel are the warmest.
• Mid-rise buildings with compact layout display high temperature.
• Areas with buildings above 10 stories tend to be cooler than agricultural lands.

## 1 Introduction

Extreme high temperatures as a result of climate change have increased in frequency, duration, and intensity, increasing the heat-vulnerable population and economic losses (Mora et al., 2017; Watts et al., 2018). The acute increase in heat exposure and vulnerability found in populated areas (Watts et al., 2018) illustrates the additional impact from the urban heat island effect (UHI), which amplifies both thermal intensity and economic losses (Estrada, Botzen, & Tol, 2017). Cities located in intertropical regions may be at higher risk of excess heat due to both global climate patterns and local development features (Giridharan & Emmanuel, 2018; Mora et al., 2017). A higher number of lethal hot days are predicted to occur in lower-latitude areas, because the combination of high humidity and warmer temperature reduces the chance of evaporative cooling (Mora et al., 2017) and the efficiency of heat convection (Zhao, Lee, Smith, & Oleson, 2014). Furthermore, intertropical cities in Asian countries face radical urbanisation (Oke, 1986); dense and compact development; and pronounced social-environmental problems such as air pollution, energy consumption, water security and public health which are related to and exacerbated by heat (e.g. Blanco, McCarney, Parnell, Schmidt, & Seto, 2011; Fan et al., 2019; Watts et al., 2018).

The characteristics of UHI are, however, not universal and vary with geographical regions (Zhao et al., 2014). Recent urban climate studies have revealed the unique pattern of UHI in warm climates (Alavipanah, Schreyer, Haase, Lakes, & Qureshi, 2018; Giridharan & Emmanuel, 2018). Yet for research at a scale that can support urban planning with scientifically informed strategies, cities in warm climates are still not as well documented as temperate cities (Giridharan & Emmanuel, 2018; Ramakreshnan et al., 2018; Shafaghat et al., 2016). Given the strong implications for urban planning and design, there are calls for greater enquiry into place-specific development patterns which shape intra-urban microclimate and heterogeneity in heat exposure within cities (Alavipanah et al., 2018; Bechtel et al., 2019; Emmanuel, 1993; Hebbert & Webb, 2012). In response, through empirical assessment of the thermal influence from urban development pattern in a specific warm climate context - Taipei - this paper provides evidence for planning cooler cities and proposes recommendations for urban planning research and practice elsewhere in response to urbanisation and warming impacts.

## 2 Background

Regardless of climate context, spatial variation of heat intensity amongst urban neighbourhoods is closely related to the proportion and spatial pattern of different land cover (Connors, Galletti, & Chow, 2013; Kong, Yin, James, Hutyra, & He, 2014; Li, Zhou, & Ouyang, 2013; Zhou, Huang, & Cadenasso, 2011; Zhou, Wang, & Cadenasso, 2017); land use (Bechtel et al., 2019; Myint et al., 2015); built-up geometry (Bechtel et al., 2019; Giridharan & Emmanuel, 2018); radiative properties of objects, and anthropogenic heat release (Bechtel et al., 2019; Oke, 1986) in both the horizontal and vertical dimensions of cities (Alavipanah et al., 2018; Tian, Zhou, Qian, Zheng, & Yan, 2019; Zheng et al., 2019).

Amongst these factors, the urban-rural temperature difference in warm climates has a closer magnitude to intra-urban temperature difference, which is mainly attributable to the distribution of vegetation (Giridharan & Emmanuel, 2018). This emphasises the vital role of greenspace planning in mitigating urban heat island effects in warm climates (Fan et al., 2019). There is limited agreement on how the efficiency of greenspace cooling effect may vary in cities depending on climate type (Zhou et al., 2017). However, greenspace cooling effect is often influenced by types of vegetation through the difference in shading and evapotranspiration rate (Skelhorn, Lindley, & Levermore, 2014); spatial configuration of greenspaces (Du et al., 2017; Shih, 2017a; Yu, Xu, Zhang, Jørgensen, & Vejre, 2018; Zhou et al., 2017); and interrelation with surrounding built environments (Li et al., 2013; Shih, 2017b; Zhao, Sailor, & Wentz, 2018; Zheng et al., 2019). Spatially, greater tree coverage, larger greenery area and higher coherence of greenspaces are a preferable structure for delivering cooling to a local scale (Li et al., 2013; Maimaitiyiming et al., 2014; Zhang, Murray, & Turner Ii, 2017). Nevertheless, both greenspace cooling intensity and extension are modifiable by the thermal state of the adjacent non-green areas (Li et al., 2013; Middel, Häb, Brazel, Martin, & Guhathakurta, 2014) and the relative location of greenery to buildings (Zhao et al., 2018). A more comprehensive cooling strategy should consider the interplay between grey and green environments (2017b, Shih, 2017b; Zhao et al., 2018; Zheng et al., 2019).

Urban climate research has provided insights into temperature influence from development characteristics of built environments. Factors related to planning decisions include building height to street width ratio (aspect ratio); building height to floor area ratio; building coverage ratio in relation to density/compactness, street/building orientation, and relative location within a building block (Ali-Toudert & Mayer, 2006; Middel et al., 2014; Pacifici, de Castro Marins, de Mello Catto, Rama, & Lamour, 2017; Shafaghat et al., 2016; Tian et al., 2019; Unger, 2006). Among these attributes, the thermal effect from building height and density may be particularly sensitive to the climate difference of cities (Alavipanah et al., 2018; Ali-Toudert & Mayer, 2006). Although greater development intensity might impede ventilation and trap long-wave radiation in narrow street canyons (Yang et al., 2019; Unger, 2006), densely distributed tall buildings do not necessarily result in greater heat intensity (Emmanuel, 1993; Zheng et al., 2019) as building shade cooling adjacent areas is particularly important for warm- to hot climate cities (Alavipanah et al., 2018; Emmanuel, 1993). In addition, development intensity determines the level of solar radiation, overshadowing (Chow & Roth, 2006; Lindberg & Grimmond, 2011), surface albedo (Giridharan, Lau, Ganesan, & Givoni, 2007), and ventilation/air flow (Gago, Roldan, Pacheco-Torres, & Ordóñez, 2013; He, Ding, & Prasad, 2019); and also closely relates to the distribution of open space and greenery, which are critical to moderate UHI.

Researchers increasingly advocate a holistic consideration of greenspaces and built environments when proposing strategies for mitigating urban heat (Shih, 2017b; Zheng et al., 2019). However, particularly in a warm climate context, the number of studies explicitly considering the integration between ‘green’ and ‘grey’ infrastructure is limited. This study responds to this gap by focusing on the spatial arrangement of buildings *and* green spaces, with a view to generating insights which can inform planning decisions.

## 3 Data and analytical methods

**Fig. 1:**
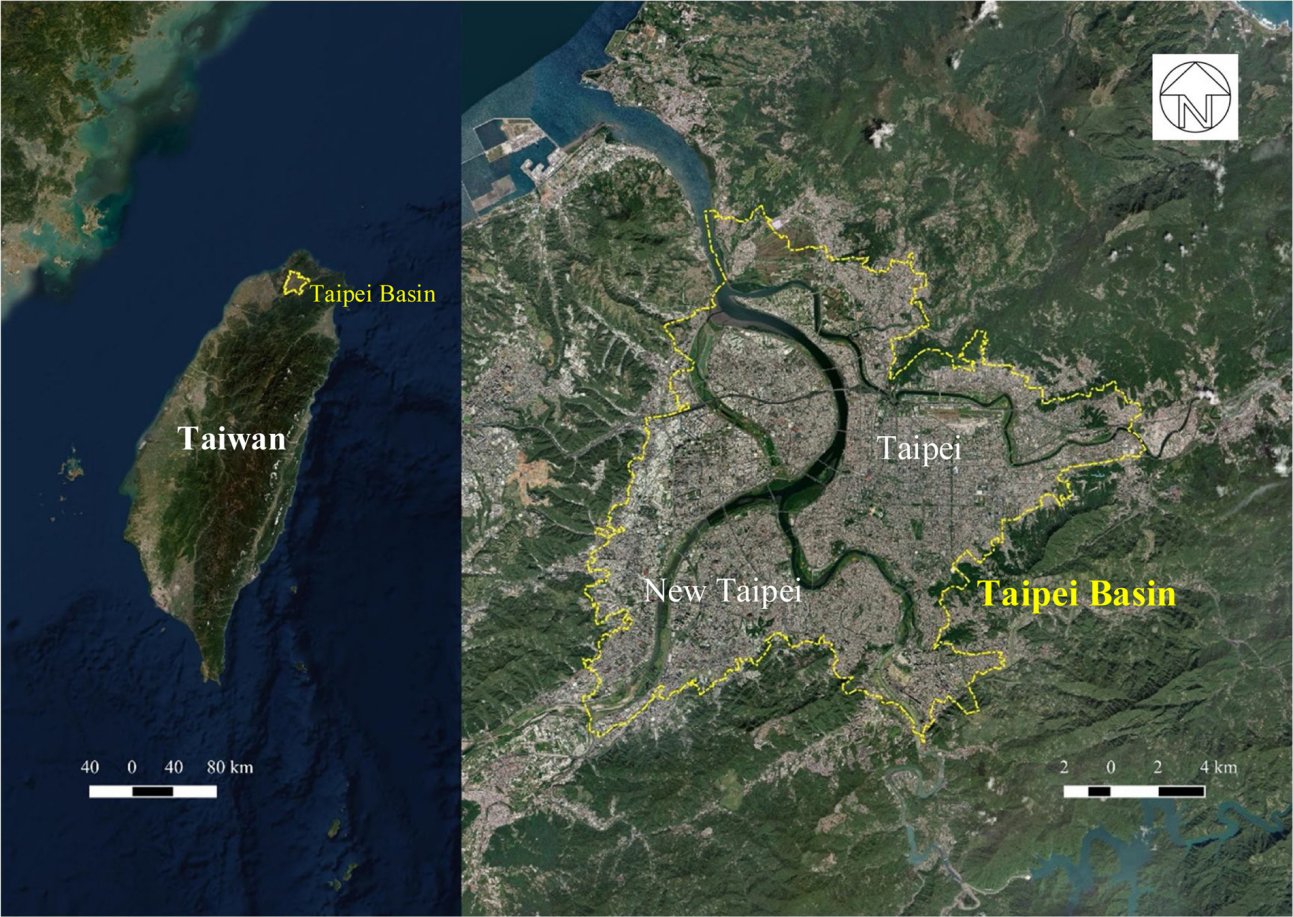
Study area – Taipei Basin (Base map from Esri Imagery).

This study takes the urbanised area in Taipei Basin (25°’N, 121°’E), which encompasses parts of Taipei City and New Taipei City, as an empirical study area (Fig. 1). Taipei Basin is about 10 km from the sea and is surrounded by forested mountains. Given its flat topography, urban expansion and densification in the past decades mainly occurred in the basin area and resulted in a compact development pattern. The boundary of the urbanised area in this study is defined by the administrative boundaries of neighbourhoods which are situated in the basin. The study area covers approximately 232.2 km^2^ and has a population estimated at 6.67 million in 2014 (Department of Household Registration, 2015). Most areas are tightly packed with three- to nine-storey buildings. High rise buildings above ten stories are generally located in new development areas at the outskirts or inside the city in urban regeneration sites. Factories, generally less than three stories high, are mainly distributed to the west of the basin in New Taipei City. The largest unbuilt land of the basin is the Quandu Plain to the North, including farmlands and wetlands. Although there is limited space for new construction, the current aim to facilitate urban regeneration in both Taipei City and New Taipei City provides an opportunity to consider how land use may be adjusted to attain better thermal environments within urban development.

The climate in northern Taiwan is humid subtropical, with an annual average temperature of about 23℃ (CWB, n.d.). The warming trend has been distinct in the urbanised basin areas. Both the intensity and frequency of hot summer days have increased significantly in the last four decades (Bai, Juang, & Kondoh, 2011; CWB, 2014; Lin, Chien, Su, Kueh, & Lung, 2017). This warming tendency places pressure on electricity networks due to rising cooling demands and may increase heat-related health problems, especially given Taiwan’s increasingly elderly population (Chen, Wu, Pan, Chen, & Lung, 2016). It is hence vital to understand how spatial variations in heat exposure arise, and how risk may be reduced through built environment planning.

### 3.1 Main sources of spatial data

This study assesses the influence of land development patterns on heat heterogeneity, employing land surface temperature and geoinformation interpreted mainly from satellite imagery. Remote sensing techniques of this nature enable citywide observation on the relationship of urban morphology and thermal distribution (e.g. Kong et al., 2014; Myint et al., 2015) and are particularly valuable for cities that are short of available meteorological data and inventory of land development patterns (e.g. Stewart & Oke, 2012; Zhou et al., 2011).

This study utilised LANDSAT 8 satellite imagery from the United States Geological Survey as principal spatial data (USGS). LANDSAT 8 visits Taiwan at 10:20 am with a 16-day interval, providing regular observations of land development patterns and daytime surface temperature. This study used multispectral data (bands 1–7) for interpreting land cover, and thermal data from Band 10 for retrieving land surface temperature as it has higher accuracy (U.S. Geological Survey (USGS), 2016). All multispectral images have a spatial resolution at 30 m. Thermal images were resampled from 100 m to 30 m. This spatial resolution allows the city-wide land-use/land-cover and thermal relationship to be observed.

Level 1 T data was applied on 6 April 2015 (spring), 29 July 2016 (summer), and 16 November 2015 (autumn). Imagery selection was based on three criteria: minimal cloud effect; high air temperature on the ground (around 30 degrees Celsius) in the Taipei region; and for the sampled days to be as close to each other as possible to avoid change in land development patterns between the images. The summer data was adopted from July 2016 instead of 2015, as the study area was mostly affected by clouds in the 2015 imagery. The image on 6 April 2015 was chosen for interpreting land development pattern, as it has the least cloud effect of all the selected images. To avoid misinterpretation of land development settings and LST from the effect of clouds, the images of Quality Band showing the location of both visible and invisible clouds were overlapped with multispectral and thermal images. Accordingly, pixels containing clouds and shadows were treated as missing values and eliminated manually through QGIS for the following analyses.

### 3.2 Deriving land surface temperature

According to the weather station of Taipei, the weather conditions on the three studied dates were warm, humid and calm (Table 1). No precipitation was recorded for each date or the day before. The mean hourly air temperature at 10 a.m. was 30.4℃, 35℃ and 29.6℃ respectively on 6 April 2015, 29 July 2016, and 16 Nov 2015. The weather condition was relatively calm, with wind speed below 1 m/s on each day. Due to minimal influence from wind on the ground, these are considered optimal conditions to assess the effect from land development patterns on LST.

**Table 1:** Meteorological conditions on the satellite acquisition date.

Dates	Meteorological record from Taipei Station		Landscape Surface Temperature		
			Mean	Min.	Max.
6 April 2015	Air temperature (℃)	30.4	29.38	21.7	34.0
	Relative humidity (%)	68	–	–	–
	Wind speed (m/s)	0.9	–	–	–
29 July 2016	Air temperature (℃)	35	32.0	24.0	36.4
	Relative humidity (%)	57	–	–	–
	Wind speed (m/s)	0.6	–	–	–
16 Nov 2015	Air temperature (℃)	29.6	25.1	20.3	30.4
	Relative humidity (%)	70	–	–	–
	Wind speed (m/s)	0.7	–	–	–

*LST was retrieved from Band 10 of Landsat 8 satellite image on each date.

Following Congedo (2016), atmospheric correction was conducted using the Dark Object Subtraction method, spectral radiance and at-satellite brightness temperature were calculated, and degrees Kelvin were converted into degrees Celsius by means of the Semi-Automatic Classification plugin on QGIS 2.18. Table 1 shows the minimum, maximum and mean LST of each date at the study areas. The LST variation was significant, reaching at least 10℃ difference on each date. To reduce potential bias deriving from the specific weather or ground conditions of one specific day, the mean value of LST data from three thermal images was used to define dependent variables at the pixel level. At neighbourhood levels, both mean and mode values of LST were applied to detect possible influence from extreme values caused by different land cover types. While some studies found a close relationship between surface and air temperature (Coppernoll-Houston & Potter, 2018; Middel et al., 2014), others suggest a more complicated relationship and variations between surface and air temperature (Shiflett et al., 2017). This difference may be caused by oversampling horizontal areas, such as rooftops, treetops, roads, and open grounds, and neglecting vertical surfaces and areas below canopy (Arnfield, 2003). We hence pay cognisance to this characteristic and its potential influence in the interpretation and discussion of our findings.

### 3.3 Interpreting development patterns

Several factors developed to assess land development pattern rely on detailed site survey and are often not comprehensive. To address this drawback, this study prioritises factors that can be interpreted from satellite images. Six factors are chosen to describe land development characteristics, including land cover, degree of greenness, greenspace proportion, greenspace coherence, built-up geometry, and primary building height (Table 2).

**Table 2:** Variables used to describe land development patterns.

Variables	Spatial Scale	Description (unit)
Land cover	Pixel	Mean NDVI of pixels
Degree of greenness (NDVI)	Neighbourhood	Mean NDVI of a given neighbourhood
Greenspace proportion (GS_ratio)	Neighbourhood	The proportion of vegetated areas of a given neighbourhood (%)
Greenspace coherence (GS_ch)	Neighbourhood	Mean shortest distance to the nearest greenspaces of a given neighbourhood (m)
Built-up geometry	Pixel	The types of Local Climate Zones
Primary building height (Pbh)	Neighbourhood	The mode value of Digital Surface Model (DSM) of a given neighbourhood (m)

#### 3.3.1 Mapping greenspaces

To define the degree of greenness and the location of greenspaces, this study applies the Normalised Difference Vegetation Index (NDVI) for detecting vegetated areas. The satellite image of 16 April 2015 was selected for calculation because it provides clear images with the least cloud coverage. The equation used for counting NDVI is:

(1) NDVI=(NIR-R)/(NIR + R),

where NIR represents near-infrared (Band 5) and R represents the red band reflectance in visible radiation (Band 4). This vegetation fraction produces a linear scale of measurement between 1 and minus 1, in which a value closer to 1 reflects greater greenness (Gandhi, Parthiban, Thummalu, & Christy, 2015). Dense vegetation such as forests and trees at a peak growth stage generally correspond to high NDVI values (approximately 0.6 to 0.9); sparse vegetation such as grasslands and scattering shrubs tend to result in moderate NDVI values (approximately 0.2 to 0.5) (Simonetti, Simonetti, & Preatoni, 2014). Barren areas, rock, and impervious surface in urban areas often result in lower value (close to 0.1) in NDVI; and water bodies are generally shown with a negative value (Gandhi et al., 2015). Because of this characteristic, NDVI is commonly used for mapping vegetation and detecting land cover (e.g. Deng et al., 2018; Thanapura et al., 2007).

Because the threshold value of NDVI for different land cover types is locally specific and the condition of vegetation varies with climate and seasonal conditions, the value used to differentiate vegetated and non-vegetated areas was determined with guides by evidence from the lead author’s previous site survey and remote-sensing based research in Taipei (reference add later). We computed NDVI with thresholds ranging from 0.2 to 0.5 and compared the result with high-resolution aerial photos from Google Earth taken in 2015. On this basis, an NDVI value of 0.4 was determined to be most representative of greenspace areas, and hence set as a threshold value to map the location of greenspaces. For validation, we randomly sampled 100 sites for accuracy assessment with aerial photos, which resulted in an accuracy rate at 76 %. Small areas of greenery, particularly street trees, often failed to be interpreted due to the lower resolution of LANDSAT satellite imagery (30 m). Greenspaces with dried lands and short plants also tended to be ignored in our mapping (see Section 5.4).

Greenspace proportion (GS_ratio) was defined by the ratio of the surface area of greenspaces to the size of a given neighbourhood. Greenspace coherence (GS_ch) was calculated on the basis of nearest neighbour distance. Using the proximity calculator function in QGIS, a proximity map was generated from the NDVI map, which subsequently assigned each pixel with a value of the shortest distance to the nearest greenspace. Using zonal statistic method, the mean proximity value of greenspace coherence of each neighbourhood was computed and extracted.

#### 3.3.2 Defining local climate zones

For interpreting built-up geometry, this study adopted the Local Climate Zones (LCZs) scheme. The LCZ scheme is a standardized and automatic method to classify the urban fabric according to climate-related properties - including sky view factor, aspect ratio, building surface fraction, impervious surface fraction, and height of roughness elements - from satellite images (Stewart & Oke, 2012; Rodler & Leduc, 2019). A Local Climate Zone is defined as a region of uniform surface cover, structure, material, and human activity that spans hundreds of meters and reflects the most prominent screen-height temperature in dry, calm, and clear weather conditions (Stewart & Oke, 2012).

As shown in Table 3, LCZs are composed of 10 “built types” (1–10) and 7 “land cover types” (A to G). Within the built types, LCZs 1–3 represent a compact layout with narrow street canyons, paved grounds and limited vegetation; LCZs 4–6 represent open layout with open street canyons, paved ground, and scattered vegetation. To reflect the local context, LCZ7 was redefined for this study as an area dominated by compact low-rise buildings, hard paved grounds, and lightweight building materials. This reflects the specific built-up fabric of light industrial areas, which are often constructed by steel huts, in the Taipei study area.

**Table 3:** The LCZ classification scheme adopted and revised from Stewart and Oke (2012)*.

* Note that LCZ7 was redefined by this study as compact low-rise areas which are hard paved and consist of light-weight building materials.

Following the World Urban Database and Access Portal Tools (WUDAPT) protocol, we firstly defined training areas for each LCZ type from northern Taiwan (to encompass LCZ types with characteristics of rural areas which may not be found in Taipei) through Google Earth Pro (7.1.5.1557) software. These training areas were then utilised as the basis for supervised classification of LCZs in the study area using SAGA GIS software (2.2.0) and the LANDSAT 8 satellite image acquired on 16 November 2015. The spatial resolution used for the LCZ classification was tested between 200 m, 100 m and 30 m. Because of significant heterogeneity in the development pattern across a relatively short distance found in Taipei, a lower spatial resolution was not deemed able to adequately reflect the nature of spatial variation found in Taipei. Considering that notable thermal variations may occur in an LCZ type when it contains greater heterogeneity (Leconte, Bouyer, Claverie, & Pétrissans, 2015; Wang, Ren, Xu, Lau, & Shi, 2018) and that the LCZ map with 30 m resolution was able to interpret more detailed spatial information, the 30 m resolution map was applied for the purposes of this study.

The accuracy of LCZ mapping relies on local knowledge from the user and the suitability of training areas. Existing research suggests LCZs provide a good indication of thermal environments with observational and numerical modelling data, but that the method itself does not provide a measure to assess mapping accuracy (Stewart & Oke, 2012). A recent accuracy assessment of the LCZ method from Ren et al. (2019) into 20 cities suggests mean accuracy can reach 76 %. To enhance the reliability of mapping, this study repeated the mapping processes and compared the results with indicative locations from aerial photos until the classification adequately reflected the development pattern. Using multiple spatial information, including the NDVI map and the Global Digital Surface Model, this study provided data triangulation for assessing building height and greenness and gave additional sources for confirming the findings from the LCZ analyses.

#### 3.3.3 Building height

As above, the second data source from the Global Digital Surface Model (DSM) dataset - ALOS World 3D-30 m (version 2.1) produced by Japan Aerospace Exploration Agency (Japan Aerospace Exploration Agency (JAXA), 2018) was applied to provide additional information for interpreting building height (Pbh). The DSM data has a horizontal resolution of approximately 30 m that was converted from the 5 m AW3D DSM dataset with a good accuracy level (<5 m) (JAXA). As the study area is covered by two images, N025E121 on the north and N024E121 on the south, from different acquisition dates, this study selected N025E121 data, which was acquired on March 2012 and covered most of the study area, for analyses. Primary building height of each neighbourhood was determined by the mode value of DSM using zonal statistic method for calculation.

### 3.4 Spatial and statistical analysis

This study used two spatial levels for analysis - pixel level and neighbourhood level. For pixel-level analysis, raster images recording the value of LST, LCZ, NDVI, and building height were exported from the QGIS software as comma-separated values (CSV) files for statistical analysis in IBM SPSS Statistics (Version 25) software. After eliminating areas containing clouds and shadows, 246,282 pixels were exported for statistical analysis. To observe the relationship between LST and greenness, a scatterplot was applied between the average LST value of three study dates and NDVI value. For examining temperature variations amongst different development types, descriptive statistics and boxplots were used to describe mean LST against LCZ types. A one-way ANOVA was conducted to compare the mean temperature of LCZ types. Further examination on the influence from building density was performed by Tukey Honestly Significant Difference (HSD) Test for post hoc comparisons amongst LCZ types. To provide further insight into the effect from greenery on built-up areas with open layout, a one-way ANOVA was measured between NDVI value (using 0.4 as threshold) and the mean temperature of three building types, namely low-rise, mid-rise, and high-rise buildings.

At the neighbourhood level, this study focuses on urbanised areas in the basin and excludes neighbourhoods that are affected by clouds. This resulted in 991 neighbourhoods across Taipei and New Taipei jurisdictions of Taipei Basin for further analysis. Utilising the zonal statistic method in QGIS, this study uses the administrative boundary of neighbourhoods to extract LST, principal LCZ types, degree of greenness, greenspace proportion, greenspace coherence, and building height for statistical analysis (Table 2). A Pearson correlation coefficient was firstly conducted to assess the strength and the direction of bivariate relationship between LST and all continuous variables of degree of greenness, greenspace proportion, greenspace coherence, and building height.

Secondly, we computed Ordinary Least Squares (OLS) regressions for examining strength and significance in temperature difference resulting from built-up geometry described by the principal LCZ types and greenspace coherence. Principal LCZ type was designed as an independent variable in a categorical form. Greenspace coherence was held constant in the regression analysis while examining temperature influence from LCZs. Conversely, LCZ type was held constant while analysing the influence from greenspace coherence, which was measured by average distance between greenspaces. In the OLS regression model, both LST and greenspace coherence were logarithmically transformed, because we found a better descriptive power for LST as the equation below:

(2) y_i_ = α + β*x_i_ + ε_i_

where y (dependent variable) is the Land Surface Temperature in ˚C (log) in observation *i*, which is determined by two explanatory variables (*x*_1_ = greenspace coherence (log), and *x*_2_ = principal LCZ type) in observation *i*, α represents intercept and β represents coefficients of the model. ε represents error in observation *i*. In this model, LCZ type is taken as categorical variable, where LCZ2 (compact mid-rise) is taken as a reference category for comparison, because it is the commonest type in the study area and is often associated with residential areas and residential and commercial mixed-use areas that are subject to warmer temperatures. To avoid possible collinearity of predictors undermining the robustness of the model, a Variance Inflation Factor (VIF) was applied between independent variables. A VIF value smaller than 5 is considered to represent no collinearity problem between independent variables (Rogerson, 2010).

## 4 Results

**Fig. 2:**
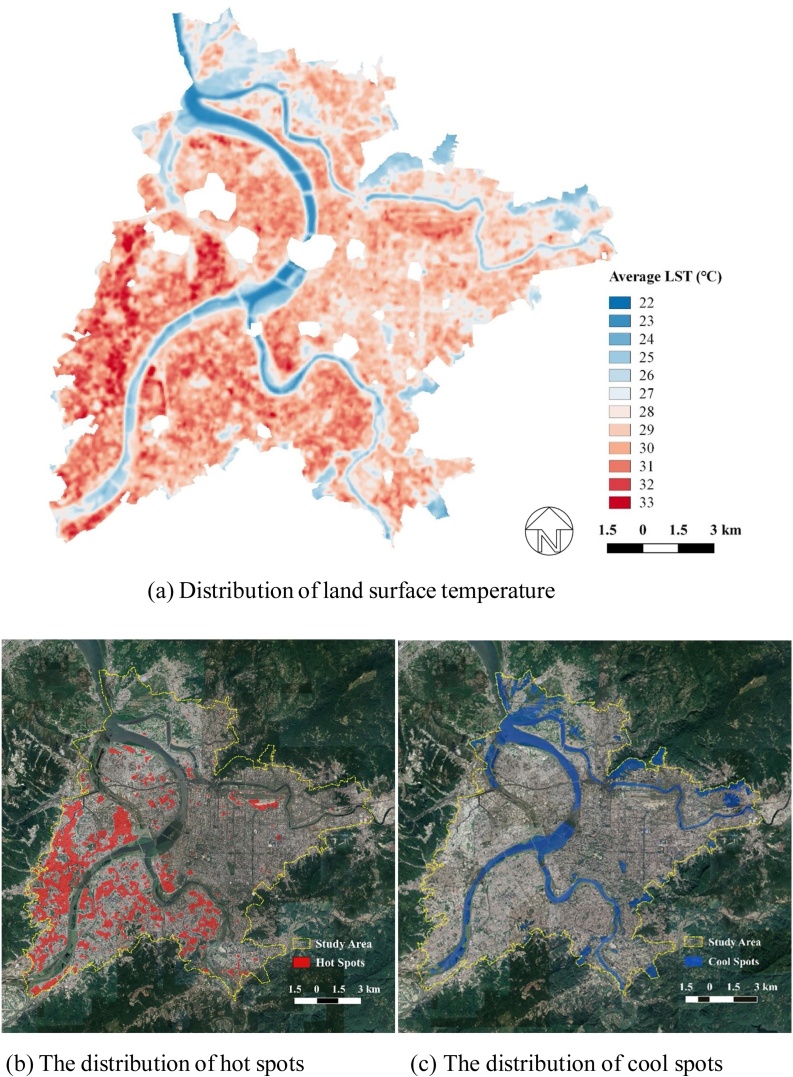
Thermal distribution in the study area.

**Fig. 3:**
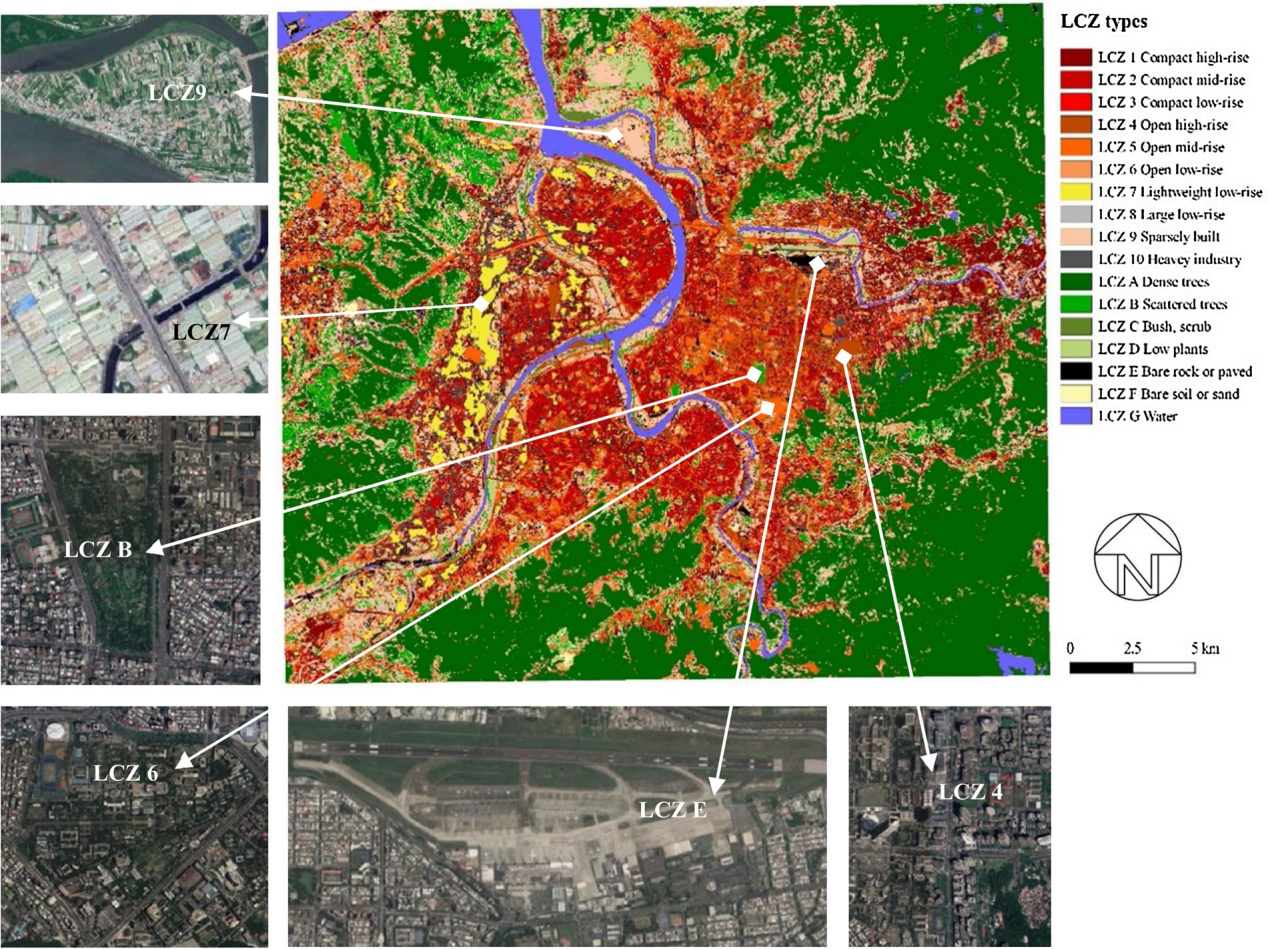
Local Climate Zones of Taipei and indicative samples of satellite image used for validation.

Fig. 2a–c provide a high-level overview of thermal distribution in the Taipei study area. The average LST in the study area ranged from 22.52℃ to 32.98℃, with a standard deviation of 1.96℃ (Fig. 2a). Hot spots were interpreted as areas one standard deviation warmer than the mean LST (Fig. 2b). Most hot spots were distributed within New Taipei City and many of them were associated with industrial areas. Cool spots, meanwhile, were interpreted as areas one standard deviation lower than the mean LST (Fig. 2c). These areas overlapped with water bodies, large parks, wetlands, farms and woodlands. Interestingly, some rooftops were also identified as cool spots, suggesting some roofing materials emit lower temperatures. As shown in Fig. 2c, although most areas cooler than the mean LST are related to waters and greenspaces, some of them are built up areas. In particular, newly developed areas in districts such as Neihu and Xinyi (Taipei City) and Banqiao (New Taipei City) were found to be cooler than the other built-up types. To understand these findings and their significance in more depth, this study further evaluated (a) influence from land cover on LST, (b) thermal influence from development intensity and built-up typology, and (c) cooling effect of greenspace configuration (Fig. 3).

### 4.1 Influence of land cover on LST

LCZ analysis reflects the spatial pattern of thermal distribution, indicating that LST varies with both land cover type and built-up geometry. Areas dominated by water and vegetation show lower temperatures than those dominated by buildings and paved grounds (Table 4). The cooling magnitude of water (LCZ G) was stronger than vegetation (LCZs A, B, C, and D), of which trees (LCZs A and B) perform greater cooling intensity than shrubs (LCZ C) and grass (LCZ D). Areas containing greater tree coverage (LCZs A and B) have lower standard deviation than those of low plant areas (LCZ D), indicating a more consistent or stable cooling effect from trees.

**Table 4:** Mean LST by types of Local Climate Zones (pixel level analysis).

LCZ types	Typical land use types in Taipei	Mean LST	N	Std. Deviation	Std. Error	Mean NDVI
1: Compact high-rise	Commercial areas	26.615	11162	0.843	0.008	0.222
2: Compact mid-rise	Residential and commercial areas	28.637	51833	0.720	0.003	0.157
3: Compact low-rise	Old development areas, burial grounds	28.312	1293	0.596	0.017	0.149
4: Open high-rise	New development areas	26.732	24023	0.911	0.006	0.224
5: Open mid-rise	Institutional grounds, school grounds	27.701	40900	0.865	0.004	0.379
6: Open low-rise	Institutional grounds, school grounds, burial grounds	26.013	1304	0.786	0.022	0.601
7: Lightweight low-rise (redefined)	Factories, industrial areas	30.223	11050	0.704	0.007	0.186
8: Large low-rise	Heavy industrial areas	27.523	1597	0.588	0.015	0.536
9: Sparsely built	Agricultural lands	26.954	40358	1.260	0.006	0.468
10: Heavy Industry	Industrial areas	28.696	16609	1.034	0.008	0.226
A: Dense trees	Woodlands, gardens, mangroves	24.298	5370	0.574	0.008	0.810
B: Scattered trees	Parks, school grounds	25.826	5649	0.581	0.008	0.719
C: Bush, scrub	Parks, burial grounds	25.065	1778	0.689	0.016	0.700
D: Low plants	Parks, riverside greenspaces, agricultural lands	26.912	3760	1.093	0.018	0.600
E: Bare rock / paved	Parking lots, airport, plaza	28.343	15665	1.045	0.008	0.157
F: Bare soil / sand	Sport grounds, shoal	27.727	614	0.730	0.029	0.217
G: Water	River, canals, lakes, ponds	23.631	13317	1.192	0.010	−0.301
Total		27.478	246282	1.717	0.003	0.276

This finding is consistent with the analysis of scatter plot between LST and NDVI at a pixel level. The lowest temperature was found at the area with negative NDVI values, which mostly corresponds to water areas. The temperature variation found with NDVI value above 0.4 is further described by the findings in vegetation types between LCZs A to D, where densely planted trees can be 2.6℃ cooler than areas covered with low plants. Conversely, the highest LSTs were measured in areas recording between 0 to 0.2 in NDVI, which are mostly associated with areas dominated by buildings and hard paved grounds. However, within this range of NDVI, the variation of LST was large, suggesting factors other than the degree of greenness also affect temperature.

### 4.2 Thermal influences from development intensity and built-up typology

The temperature variation caused by development intensity and built-up types was further explained by analysis with LCZs. A one-way ANOVA shows a significant effect of LCZ type on LST (F(16, 246265) = 35446.603, *p* < 0.001) (Table 5).

**Table 5:** Result from one-way ANOVA between mean LST and all LCZ types.

	Sum of Squares	df	Mean Square	F	Sig.
Between Groups	506017.32	16	31626.08	35446.60	0.000
Within Groups	219721.96	246265	0.89		
Total	725739.28	246281			

The model suggests that a 10 % increase in distance amongst greenspaces will lead to the increase of LST by 0.26 % when LCZ types were held constant. As mentioned earlier, LCZ 2 (compact mid-rise) was taken as a reference temperature to compare with other LCZ types at the neighbourhood level by OLS regression analysis. Within built-up neighbourhoods, areas dominated by compact high-rise buildings (LCZ 1) can be 4.3 % cooler than compact mid-rise areas; whereas open high-rise and open mid-rise have 4.0 % and 1.2 % respectively lower temperature in comparison to compact mid-rise (LCZ 2), when greenspace coherence is held constant (Table 6). In other words, although reducing building density (e.g. LCZ 5: open mid-rise and LCZ 9: sparely built) shows significant temperature reduction, the increase in development intensity in the vertical dimension can also lead to lower temperature (LCZ 1 and LCZ 4). Our model does not find multicollinearity between predictors, as the variance inflation factor (VIF) values were below 2 (Table 6).

**Table 6:** Result of OLS regression on LST with principal LCZ types and greenspace coherence at neighbourhood level (Dependent variable is log LST in ˚C).

Variables	Coefficient	Standard Errors	VIF
Log (greenspace coherence)	0.026***	(0.002)	1.92
LCZ 1: Compact high-rise^α^	−0.043***	(0.004)	1.08
LCZ 4: Open high-rise^α^	−0.040***	(0.003)	1.11
LCZ 5: Open mid-rise^α^	−0.012***	(0.003)	1.46
LCZ 6: Open low-rise^α^	−0.014	(0.023)	1.01
LCZ 7: Lightweight low-rise^α^	0.029***	(0.005)	1.03
LCZ 9: Sparsely built^α^	−0.033***	(0.003)	1.54
LCZ 10: Heavy Industry^α^	0.020***	(0.007)	1.02
LCZ A: Dense tree^α^	−0.066***	(0.008)	1.14
LCZ B: Scattered tree^α^	−0.056***	(0.017)	1.03
LCZ E: Bare rock / paved^α^	−0.004	(0.017)	1.00
LCZ G: Water^α^	−0.127***	(0.005)	1.02
Constant	3.274***	(0.009)	
Observations	984		
R^2^	0.670		
Adjusted R^2^	0.660		
Residual Std. Error	0.023 (df = 971)		
F Statistic	164.150*** (df = 12; 971)		

*Note:*^α^ reference: compact mid-rise (LCZ2); *p < 0.1; **p < 0.05; ***p < 0.01.

#### 4.2.1 Development density

**Fig. 4:**
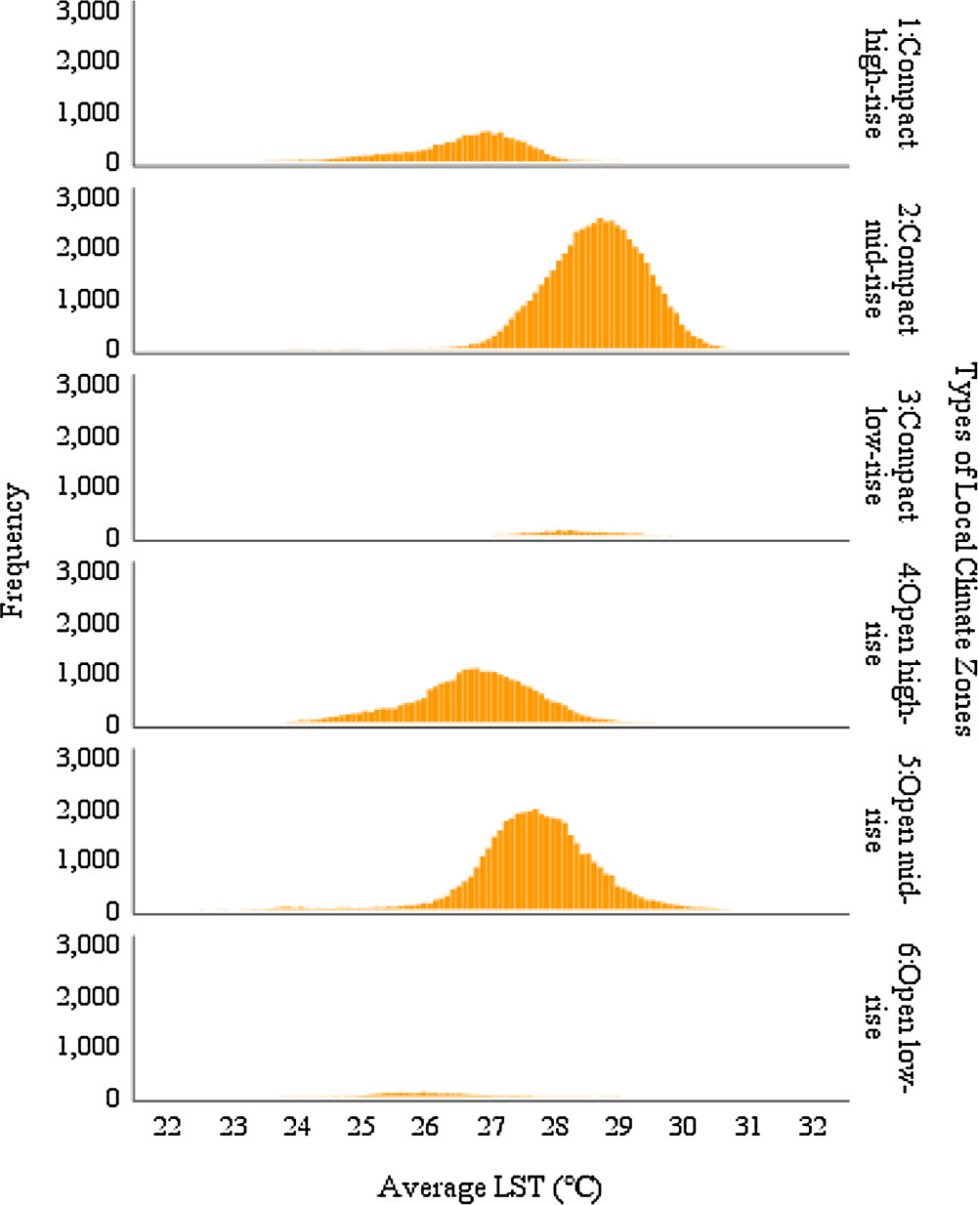
Thermal distribution between LCZ 1 to LCZ 6 at a pixel level.

The influence of building density on temperature was further observed at pixel level through the post-hoc Tukey HSD Test. The significant difference between the mean LST of LCZs 1 and 4; LCZs 2 and 5; and LCZs 3 and 6 indicates that greater openness between buildings leads to lower temperature amongst low- to mid- rise buildings (Table 7; Fig. 4). The temperature difference was particularly distinct at areas with low-rise buildings, where open layout (LCZ 6) is on average 2.3℃ cooler than a compact layout (LCZ 3). Amongst high-rise buildings, however, open high-rise (LCZ 4) was marginally warmer (0.12℃) than its compact counterpart (LCZ 1). This suggests an interactive effect from building height and spacing on temperature. Hence, reducing building density without considering other factors is unlikely to produce cooler environments for all building types.

**Table 7:** Multiple comparisons with Tukey HSD test between 1 to 6 LCZ types.

LCZ Types		Mean Difference	Std. Error	Sig.	95 % Confidence Interval	
					Lower Bound	Upper Bound
1: compact high-rise	2	−2.021*	.009	<0.001	−2.046	−1.997
	3	−1.696*	.024	<0.001	−1.765	−1.628
	4	−.117*	.009	<0.001	−.144	−.091
	5	−1.085*	.009	<0.001	−1.110	−1.061
	6	.602*	.024	<0.001	.534	.670
2: compact mid-rise	1	2.021*	.009	<0.001	1.997	2.046
	3	.325*	.023	<0.001	.260	.391
	4	1.904*	.006	<0.001	1.886	1.923
	5	.936*	.005	<0.001	.921	.952
	6	2.624*	.023	<0.001	2.559	2.689
3: compact low-rise	1	1.696*	.024	<0.001	1.628	1.765
	2	−.325*	.023	<0.001	−.391	−.260
	4	1.579*	.023	<0.001	1.513	1.646
	5	.611*	.023	<0.001	.545	.677
	6	2.299*	.032	<0.001	2.208	2.390
4: open high-rise	1	.117*	.009	<0.001	.091	.144
	2	−1.904*	.006	<0.001	−1.923	−1.886
	3	−1.579*	.023	<0.001	−1.646	−1.513
	5	−.968*	.007	<0.001	−.987	−.949
	6	.719*	.023	<0.001	.653	.785
5: open mid-rise	1	1.085*	.009	<0.001	1.061	1.110
	2	−.936*	.005	<0.001	−.952	−.921
	3	−.611*	.023	<0.001	−.677	−.545
	4	.968*	.007	<0.001	.949	.987
	6	1.688*	.023	<0.001	1.622	1.753
6: open low-rise	1	−.602*	.024	<0.001	−.670	−.534
	2	−2.624*	.023	<0.001	−2.689	−2.559
	3	−2.299*	.032	<0.001	−2.390	−2.208
	4	−.719*	.0232	<0.001	−.785	−.654
	5	−1.688*	.023	<0.001	−1.753	−1.622

Notes: * The mean difference is significant at the 0.05 level.

**Fig. 5:**
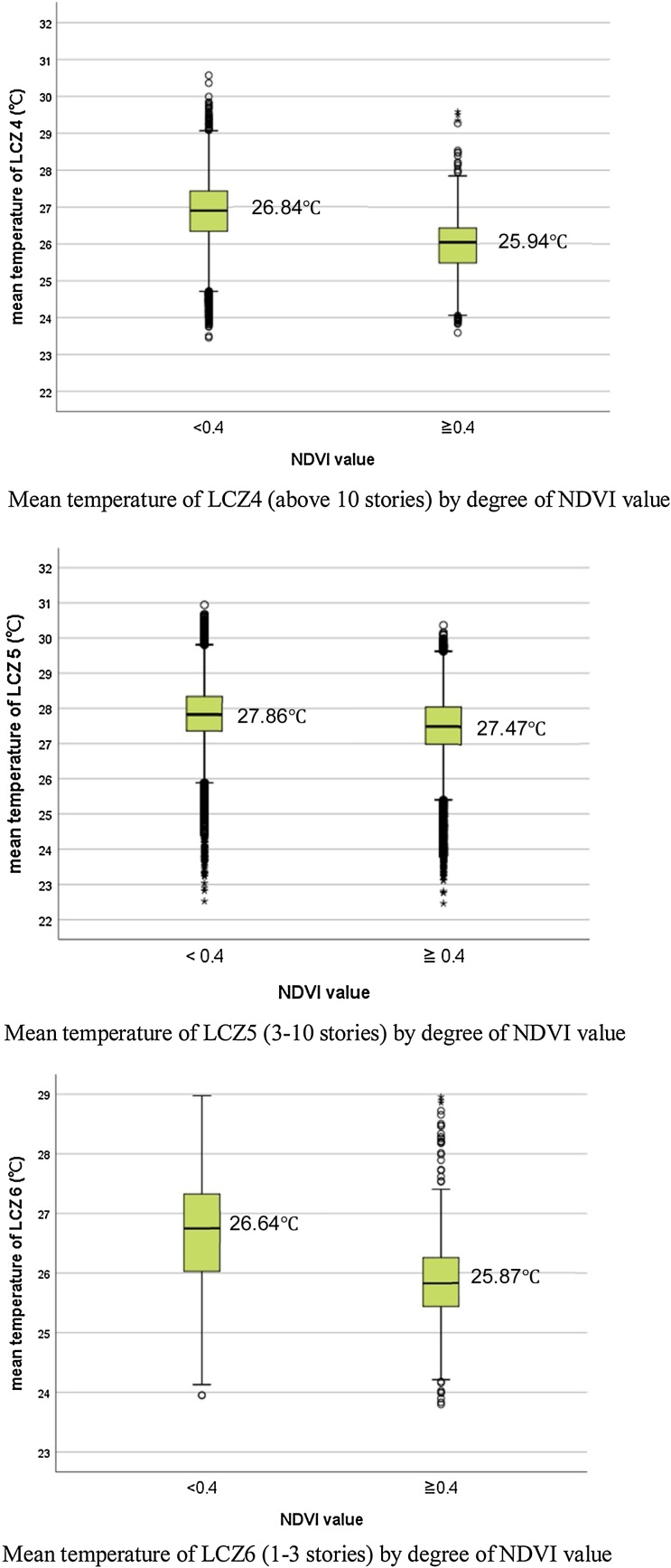
Mean LST of three types of buildings located in an open layout against NDVI value.

Likewise, the lower temperature found with an open layout is not only caused by the increase of spacing, but also by greater greenery in the surroundings. As Table 4 shows, both LCZs 5 and 6 have greater greenness than their compact counterparts. To compare the effect from greenness, a one-way ANOVA and boxplots were applied to the mean temperature of LCZs 4, 5, and 6. The results show significant lower temperature for all building types when the NDVI value was greater than 0.4 (Fig. 5). The temperature difference between areas with and without greenery reached 0.91℃ in LCZ4 (F(1, 24021) = 2890.55, *p* < 0.001); 0.38℃ in LCZ5 (F(1, 40898)=2001.02, *p* < 0.001); and 0.78℃ in LCZ6 (F(1, 1302)=223.02, *p* < 0.001). The lower cooling intensity found with open mid-rise buildings reflects the findings at neighbourhood level (Table 7), suggesting that greenspace cooling effect may be particularly constrained in areas surrounded by buildings with 3–10 storeys.

#### 4.2.2 Building height

**Fig. 6:**
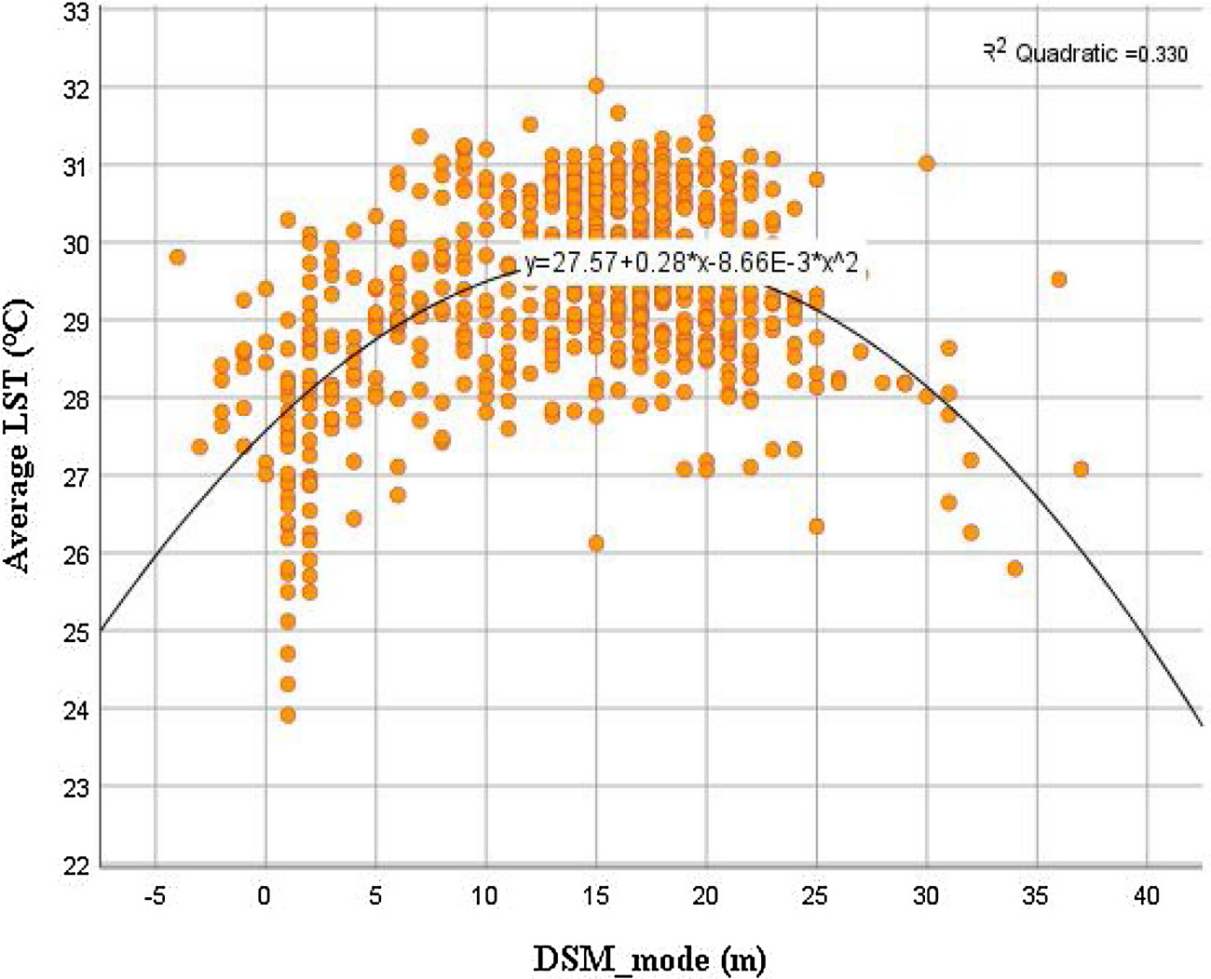
Thermal distribution against building height.

The comparison between the mean LST of LCZs 1–6 with post-hoc Tukey HSD Test at pixel level demonstrated significant temperature difference between compact built-up type from LCZs 1–3, as well as between open built-up types from LCZs 4–6. Yet regardless of compact or open layout, high-rise buildings (LCZ 1 and LCZ 4) tend to result in lower temperature, whereas mid-rise buildings (LCZ 2 and LCZ 5) tend to result in higher temperature. To triangulate data and enhance the reliability of our results, the relationship between mean LST and the majority of building height within a neighbourhood level was further examined using Digital Surface Model data. A significant quadratic relationship (R^2^ = 0.33, F(2, 709) = 174.37, *p* < 0.001) was found, confirming neighbourhoods dominated by mid-rise buildings tend to be warmer than those dominated by either low-rise or high-rise buildings (Fig. 6).

### 4.3 Effect from greenspace pattern

**Fig. 7:**
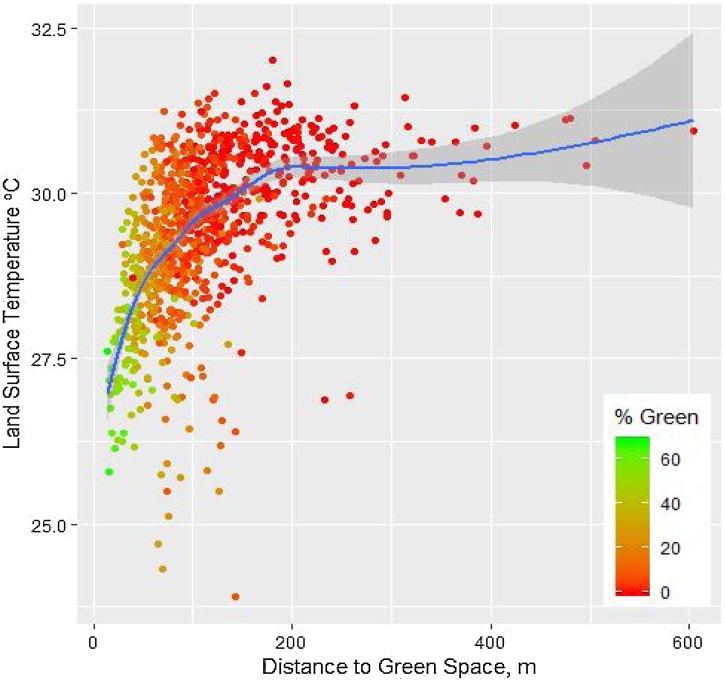
Relationship between greenspaces and LST at neighbourhoods.

At the neighbourhood level, greenspace pattern in addition to overall degree of greenness plays an important role in determining LST difference. The Pearson Correlation Coefficient analyses show that both mean and mode value of LSTs decrease with greenspace proportion (r=−0.632, p < 0.01; r=−0.203, *p* < 0.01) and increase with greenspace coherence (defined as shortest distance to nearby greenspaces) (r=0.518, *p* < 0.01; r=0.200, *p* < 0.01), at a significant level (Table 8). Greater greenspace coverage and coherence result in cooler environments within a neighbourhood. A simple linear relationship was found between mean LST and greenspace proportion of neighbourhoods (F(1, 982) = 653.126, -*p* < 0.001) with an *R*^2^ of 0.399. For every 1 % of greenspace increase in a neighbourhood, mean LST decreased by 0.05℃. Similarly, a significant logarithmic relationship was observed with greenspace coherence (F(1, 982) = 581.855, *p* < 0.001) with an *R*^2^ of 0.372. This suggests every metre increase in inter-greenspace distance is associated with a 1.18℃ rise in mean LST in a neighbourhood. As displayed in Fig. 7, the most notable cooling effect occurs in neighbourhoods where average distance between greenspaces is estimated to below 170 m. Greenspaces distributed at greater distance than this might result in little cooling effect to a neighbourhood.

**Table 8:** Pearson correlation between temperatures and greenspace features.

Greenspace features Temperature		Degree of greenness (NDVI)	Greenspace proportion	Greenspace coherence
Mean LST	Pearson Correlation	−.486**	−.632**	.518**
	Sig. (2-tailed)	.000	.000	.000
	N	984	984	984
Mode LST	Pearson Correlation	−.120**	−.203**	.200**
	Sig. (2-tailed)	.000	.000	.000
	N	991	991	991

** Correlation is significant at the 0.01 level (2-tailed).

## 5 Discussion and implications

Overall, the results of this study reaffirm that significant thermal variation can exist within the same urbanised area. Some neighbourhoods are subject to higher heat intensity than others, due to joint effect from the layout of buildings and greenspaces. Our findings show that cooler environments are not only associated with natural surfaces, but also their interrelation with different forms and spatial arrangements of buildings (Middel et al., 2014). Accordingly, we draw out conceptual insights in three areas - development intensity; greenspace configuration; and building materials and land use. Given the need for more in-depth knowledge of urban thermal environments in dense subtropical Asian cities, attention is paid to comparison with existing Asian studies.

### 5.1 Implications for development intensity: density, height, and greenness

Existing research into development intensity and thermal environments has produced varying results, even within similar climate contexts (e.g. Guo, Zhou, Wu, Xiao, & Chen, 2016; Yang et al., 2019). Our results show that an open layout tends to reduce temperature at low- to mid-rise building areas, whereas a compact layout is the coolest development form for high-rise buildings. This finding is partly inconsistent with previous studies, which have indicated an increase in temperature with building density (e.g. Chen, Lin, & Lin, 2017; Shi, Lau, Ren, & Ng, 2018; Wong & Yu, 2005), building height (Yang et al., 2019) and street openness (Ali-Toudert & Mayer, 2006) in hot to warm climates. Previous research has also observed higher temperature at densely developed high-rise buildings owing to lower ventilation efficiency (Yang et al., 2019); or at openly distributed low-rise buildings due to increase of incoming solar radiation heating up street canyons (Ali-Toudert & Mayer, 2006; Shi et al., 2018; Thorsson, Lindberg, Björklund, Holmer, & Rayner, 2011). In our study, increasing spacing was an important factor for lower temperature amongst low to mid rise buildings. This may be due to greater abundance of greenery in an open layout. As greenery (particularly tree canopy in a tropical climate) often increases with available open space in Taipei, it is likely to provide better shading and ventilation to an area.

The bell-shaped relationship found with building height and LST supports findings from Beijing, China (Feng & Myint, 2016), Guangzhou, China (Guo et al., 2016) and Phoenix, USA (Middel et al., 2014), indicating that regardless of density, high-rise buildings were the coolest type during daytime in a warm climate. The average temperature of high-rise buildings is nearly as low as sparely-built agricultural lands in Taipei, suggesting a weak intensity of urban heat island effect. As cooler environments of high-rise buildings are likely attributable to shade from surrounding buildings (Middel et al., 2014; Pacifici et al., 2017; Zheng et al., 2019), the higher solar altitude in subtropical regions - which reduces the shading efficacy of lower buildings - may explain the higher temperature found at low- to mid-rise buildings in Taipei (Oke, 1986).

Although our results regarding lower daytime temperature at high-rise building areas are consistent with previous studies, the potential difference of diurnal temperature requires further attention (Tian et al., 2019). In some cases, previous research into both daytime and night-time urban heat island effect has observed higher nocturnal temperature at compact high-rise neighbourhoods (e.g. Chow & Roth, 2006; Shi et al., 2018). Other studies based on air temperature in Taipei (Chen, Lo, Shih, & Lin, 2019) or surface temperature in Shanghai (Yang et al., 2019) have however suggested temperature increase with both building density and height. These differences in findings may be influenced by rooftop characteristics, such as size, aspect, and materials (Zhao, Myint, Wentz, & Fan, 2015); building orientation and height affecting shading and ventilation (e.g. Peng et al., 2018); dynamic traffic conditions influencing air temperature on the ground (Cardoso & Amorim, 2018); or the definition of building height taken in a particular study. Hence, a comparative study would be of value to rule out potential variables derived from the difference in methods. Further examination in relation to the time at which thermal data was collected, and the methods through which the data was collected, is therefore necessary to provide a more comprehensive overview.

### 5.2 Cooling variation from vegetation

The multiple methods of NDVI value, LCZ types, and greenspace configuration utilised in this study provide further insights into the argument that vegetated grounds are cooler than hard-paved and building areas. As per previous research in Nanjing, China (Kong et al., 2014) and Arizona, USA (Myint et al., 2015), cooling intensity in Taipei was stronger with trees yet weaker with low plants such as grass, turf and crops. Both average temperature and variation was greater at low plant areas (LCZ D). This may be explained by weak transpiration cooling from vegetation and greater openness, which subject the area to greater influence from solar exposure and shading from surroundings. Notably, some low plant areas were even warmer than some building areas. Increasing greenery without considering this characteristic may therefore undermine the effectiveness of heat reduction strategies.

Spatial distribution of greenspaces also plays an important role in determining cooling performance. This study confirms the findings of previous research suggesting greater greenspace proportion/coverage can result in lower temperature (e.g. Yu, Guo, Zeng, Koga, and Vejre (2018)) on Fuzhou, China). We further suggest that the cooling effect from increasing greenspace proportion might be limited, such that every 1% of greenspace increase within a neighbourhood leads to average temperature decrease of only 0.05℃. However, this finding does not discourage the increase of greenery. Instead, what our findings indicate is that planning greenery for cooling should consider spatial pattern as well as increasing quantity. Although previous studies have varying opinions regarding the effect from greenspace configuration on cooling (Kong et al., 2014; Shih, 2017a), our results support the viewpoint of Myint et al. (2015) and Shih (2017a) suggesting the clustering pattern is superior to scattering pattern in reducing average temperature. Greater temperature reduction was found when greenspaces were allocated close together within an estimated threshold distance around 170 m, reflecting the phenomenon of cooling decay highlighted in recent research (e.g. Lin, Yu, Chang, Wu, & Zhang, 2015; Shih, 2017b).

In addition, as Shih and Mabon (2018) argue, the intensity of cooling effect is influenced by surrounding development characteristics. Although increasing greenery with open space leads to lower temperature across all building types, the relatively small cooling intensity amongst mid-rise buildings (three to ten storeys) needs further attention. It is likely that this building height benefits less from shading but accumulates more anthropogenic heat which offsets the cooling effect from vegetation. Considering that greenery cooling effect varies with the interrelation between greenspace pattern and built-up geometry, greening strategies focusing on cooling should be tailored according to different development typologies.

### 5.3 Associations with building materials and land use

LCZ analysis suggests an association between land use type and thermal characteristics. The warmest areas were mostly related to factories and industrial areas, consistent with observations in Singapore (Chow & Roth, 2006; Jusuf, Wong, Hagen, Anggoro, & Hong, 2007). Whilst greater impervious surface is one explanation for higher temperature in industrial areas, building materials commonly used for constructing factories in Taipei have a notable influence on this thermal property (Oke, 1986). Given its lower price, many small factories in Taipei are constructed with corrugated iron sheets, which tends to absorb heat and exacerbate urban heat island effects (Wang, Chang, & Chu, 2007). This thermal characteristic is reflected by LCZ7, which is 2.7℃ above average temperature in this study.

Similarly, the common use of these iron sheets in Taiwan as roofing materials in low- to mid-rise buildings when constructing an additional room on the rooftop might to some extent contribute to heat absorption. As this rooftop construction is observed less on high-rise buildings, the higher temperature found with low- to mid-rise building areas in our study should be viewed in light of this characteristic, which is specific to Taipei and Taiwan. This is particularly important when comparing the outcomes from this study to other subtropical to tropical Asian cities. Methodologically, this insight illustrates the value of local contextual knowledge in interpreting and explaining outcomes from LCZ analysis of this nature.

### 5.4 Research limitations

Although satellite imagery enables simultaneous analyses of thermal and landscape relationship throughout a city, it has some inherent restrictions. LANDSAT 8 satellite visits study areas during daytime with a 16-day interval, so it only allows an observation in specific time of a day (10:20 a.m. in case study area) and is not able to address diurnal change. Caution should thus be exercised in generalising our results to other times of the day. In addition, the use of LANDSAT 8 imagery for interpreting greenspaces will inevitably exclude vegetation with a small surface area, due to the limit in spatial resolution of 30 m. The NDVI value used to map vegetated areas in this study also tends to mis-classify greenspaces which have dried grounds and short plants as non-green areas. Although these types of greenspaces have been demonstrated in previous research to deliver limited cooling effect due to the lack of evapotranspiration cooling (REF), it is important to acknowledge these potential influences on our classification schemes.

## 6 Conclusions

This paper broadly reinforces extant empirical research in subtropical to tropical climate contexts, indicating that land surface temperature is jointly affected by vegetation and built-up geometry. A notable finding is that high-rise buildings, regardless of open or compact layout, display lower temperatures than agricultural lands. This implies that intensive development in the vertical dimension does not necessarily lead to strong urban heat island effect and could be a development solution for reducing daytime tropical heat.

Moreover, fitting with much research to date, mid-rise building areas appear to be the warmest types within residential and commercial areas, whilst both increasing spacing or changing building height can have significant temperature reduction. The Taipei results illustrate, however, that any cooling benefits from open layouts must be balanced with greenery – especially given that close distribution of greenspaces is needed to maximise cooling benefits. It is therefore important to take both open space and vegetation into account when considering cooling strategies for reducing heat accumulation amongst low- to mid- rise buildings. This is especially significant in Taipei, where compact mid-rise buildings are the dominant development type and have resulted in highest temperatures.

Furthermore, the Taipei study shows the importance of bearing in mind local, context-specific factors when understanding thermal distribution. For instance, areas dominated by compact low-rise and lightweight factories display the highest surface temperature. This is likely attributable to not only the lack of greenery, but also the common building materials of corrugated iron steel used in Taiwan. This study also indicates a need for further empirical research across a breadth of contexts to more fully understand diurnal difference in relation to shading benefit.

Based on the findings of this paper, we suggest prioritising heat mitigation interventions to industrial areas and mid-rise building areas. Four strategies are proposed for cooling Taipei at daytime in summer: 1) increasing the amount of water bodies and vegetation, with greater coverage and coherence; 2) taking building height and shadow into account during regeneration/development; 3) increasing spacing and greenery between low- to mid-rise buildings; and 4) avoiding construction of compact low-rise buildings with corrugated iron steel. These actions could be realised by integrating findings and subsequent guidance on built environment characteristics for lower temperatures into urban regeneration plans, building codes, and urban/landscape design. Whilst context specific, further research may wish to assess the value of such strategies at night time and compare with other Asian city contexts. Caution should be also paid to other environmental impacts, such as energy use, air pollution and ventilation, derived from these cooling strategies, so as to gain a more comprehensive understanding of how to enhance the climate resilience of subtropical cities.

## Declaration of Competing Interest

The authors declare that they have no known competing financial interests or personal relationships that could have appeared to influence the work reported in this paper.
